# lncRNA *Hnscr* Regulates Lipid Metabolism by Mediating Adipocyte Lipolysis

**DOI:** 10.1210/endocr/bqad147

**Published:** 2023-10-03

**Authors:** Yi-Fan Guo, Jing-Yi Sun, Ya Liu, Zhe-Yu Liu, Yan Huang, Yuan Xiao, Tian Su

**Affiliations:** Department of Endocrinology, Endocrinology Research Center, Xiangya Hospital of Central South University, Changsha, Hunan 410008, China; Department of Geriatrics, Xiangya Hospital, Central South University, Changsha, Hunan 410008, China; Department of Endocrinology, Endocrinology Research Center, Xiangya Hospital of Central South University, Changsha, Hunan 410008, China; Department of Endocrinology, Endocrinology Research Center, Xiangya Hospital of Central South University, Changsha, Hunan 410008, China; Department of Endocrinology, Endocrinology Research Center, Xiangya Hospital of Central South University, Changsha, Hunan 410008, China; Department of Endocrinology, Endocrinology Research Center, Xiangya Hospital of Central South University, Changsha, Hunan 410008, China; Department of Endocrinology, Endocrinology Research Center, Xiangya Hospital of Central South University, Changsha, Hunan 410008, China; National Clinical Research Center for Geriatric Disorders, Xiangya Hospital of Central South University, Changsha, Hunan 410008, China

**Keywords:** obesity, *Hnscr*, lipolysis, adipocyte, lipid metabolism

## Abstract

Obesity is a process of fat accumulation due to the imbalance between energy intake and consumption. Long noncoding RNA (lncRNA) *Hnscr* is crucial for metabolic regulation, but its roles in lipid metabolism during obesity are still unknown. In this article, we found that the expression of *Hnscr* gradually decreased in adipose tissues of diet-induced obese mice. Furthermore, the deletion of *Hnscr* promoted an increase in body weight and adipose tissue weight by upregulating the expression of lipogenesis genes and downregulating lipolysis genes in inguinal white adipose tissue (iWAT) and brown adipose tissue. In vitro knockdown of *Hnscr* in adipocytes resulted in reduced lipolysis of adipocytes. Overexpression of *Hnscr* by adenovirus or drug mimics showed the opposite. Mechanistically, *Hnscr* regulated adipose lipid metabolism by mediating the cyclic adenosine monophosphate/protein kinase A signaling pathway. This study identifies the initial characterization of *Hnscr* as a critical modifier that regulates lipid metabolism, suggesting that lncRNA *Hnscr* is a potential target for treating obesity.

The worldwide obesity pandemic has dramatically increased the incidence of type 2 diabetes, cardiovascular disease, and other related complications (1, 2). Obesity is caused by excess energy intake over energy expenditure in adipocytes, stored in white adipose tissue as triglycerides (3). Lipolysis and lipogenesis maintain a dynamic balance in adipocytes (4, 5). Studies have shown that hormone-sensitive lipase (HSL) and adipose triglyceride lipase (ATGL) are essential in triglyceride catabolism (6, 7). The activity of lipases largely depends on their interaction with cofactors. HSL is a downstream target of protein kinase A (PKA). Several studies have shown that HSL can be upregulated by activating PKA phosphorylation, thereby promoting lipolysis and browning (8‐10). Different phosphorylation sites also regulate the activity of ATGL lipase (11). For instance, β-adrenergic activation can lead to PKA-mediated phosphorylation of ATGL Ser(406) to modestly increase ATGL-mediated lipolysis (12). Although some research has been conducted on lipases, much remains to be said about the function of these lipases in vivo and their relative contribution to adipocyte lipolysis.

Long noncoding RNAs (lncRNAs), defined as nonprotein-coding RNA transcripts longer than 200 nucleotides, are emerging as crucial regulators of different cellular processes (13). Accumulating evidence has revealed that lncRNAs regulate lipolysis (14‐18). lncRNA NONMMUT096150.1 modulates the adipogenesis of bone marrow mesenchymal stem cells via lipolysis regulation in adipocyte and adipocytokine signaling pathways (19). lncRNA lncIMF4 controls the adipogenesis of porcine intramuscular preadipocytes through attenuating autophagy to inhibit lipolysis (18). Alvarez et al showed that the downregulation of lncRAP2 hindered fat cell lipolysis (20). However, the mechanism of action of lncRNAs in regulating lipid metabolism needs to be investigated more.

The cyclic adenosine monophosphate (cAMP)/PKA pathway is the most well-known mechanism that mediates lipolysis (21). Increased levels of cAMP can lead to the activation of cAMP-dependent PKA. HSL is phosphorylated by PKA, resulting in the catalytic breakdown of triglycerides and diglycerides, producing free fatty acid and glycerol (21). In addition, cAMP/PKA mediates PGC-1α activation by activating ATGL, promoting oxidative metabolism in cell and animal models (22). Our previous study also indicated that RCN2 stimulates lipolysis by binding to NRP-2 and ITGB1 receptor complexes and activating the cAMP/PKA signaling pathway (23). Nevertheless, the role of lncRNA in this pathway is unclear and requires further investigation.

Our previous studies discovered that lncRNA *Hnscr* (Gm31629) is crucial to glucose metabolism (24). However, the role of *Hnscr* in regulating lipid metabolism has yet to be elucidated. In this article, we focus on the relationship between *Hnscr* and adipose tissue lipid metabolism. Decreased *Hnscr* expression in inguinal white adipose tissue (iWAT) and brown adipose tissue (BAT) was observed in high-fat diet (HFD)–induced obese mice. In in vivo and in vitro experiments, *Hnscr* reduction promotes adipogenesis and reduces lipolysis in adipocytes. In comparison, overexpression of *Hnscr* or supplementation with theaflavin 3-gallate (TF2A) stimulated adipocyte lipolysis. Mechanistically we found that *Hnscr* regulated lipid metabolism by mediating the cAMP/PKA signaling pathway. Taken together, our findings highlight *Hnscr* as a target for treating obesity by alleviating lipid metabolism disorders.

## Materials and Methods

### Animals

Eight-week-old male C57BL/6J mice were obtained from the Hunan SJA Laboratory Animal Company. Male *Hnscr*-null mice were generated as described previously (25). Briefly, to generate *Hnscr*-deficient (*Hnscr*-null) mice, the genomic region of the mouse Gm31629 gene was knocked out by using TetraOne technology from Cyagen Biosciences (China). *Hnscr* genotyping was determined by extracting genomic DNA from the toes or tails of newborn mice and performing polymerase chain reaction (PCR) with the following PCR primers: forward 1:5′-GCTAAGCTCGAGGGACCTAATA-3′, forward 2:5′-CTGCTGCTCACCTCTAGTCATT-3′, reverse: 5′-AGGGATGTTCCTCACTGGCTGG-3′.

All mice were kept in a C57BL/6J background, maintained in the specific pathogen-free (SPF) class at the Experimental Animal Research Center of Central South University, with a constant temperature of 22 to 24 °C, constant humidity of 60% to 75%, 12-hour dark/light cycle, 4 or 5 mice per cage, free access to food and water, and regular change of bedding. All animal care protocols and experiments were reviewed and approved by the Animal Care and Use Committee of the Laboratory Animal Research Center, Xiangya School of Medicine, Central South University. The study complied with all relevant ethical regulations for animal research.

### Animal Treatment

Four groups of male C57BL/6J mice, including wildtype (WT) normal chow diet (NCD) group, n = 6), WT HFD group (n = 6), *Hnscr*-null NCD group (n = 6), and *Hnscr*-null HFD group (n = 6), were fed rodent chow diet or HFD (60% kcal fat, 20% kcal carbohydrates, and 20% kcal protein, D12492; Research Diets, Wuhan BIOPIKE Bioscience Co. Ltd., Wuhan, China) for 3 months at 8-10 weeks age. Subsequently, mice were executed, and the tissues were isolated. Tissues were weighed, snap-frozen in liquid nitrogen, and then stored at −80 °C for subsequent studies.

### Cell Culture

Male WT mice were euthanized at 8 weeks of age, and stromal vascular fractions (SVFs) from iWAT pads were isolated as described in previous studies (26, 27). SVFs were grown in Nutrient mix F-12 (Dulbecco’s modified Eagle’s medium [DMEM]/F12) containing 10% fetal bovine serum (Gibco) and 1% antibiotic/antimycotic solution (Gibco, Grand Island, NY). The 3T3-L1 cells were grown in DMEM) containing 10% fetal bovine serum and 1% antibiotic/antimycotic solution. All cells were maintained at 5% CO_2_ and 37 °C. After primary iWAT adipocytes or 3T3-L1 cells reached confluence (day 0), differentiation of the cells was induced in DMEM high-glucose medium supplemented with 0.5 mM isobutyl methylxanthine (Sigma, Darmstadt, Germany), 1 μM dexamethasone (Sigma), 10 μg/mL insulin (Sigma), and 1 μM rosiglitazone (Sigma) for 3 days and then the medium changed to 10 μg/mL insulin and 1 μM rosiglitazone every 2 days until day 8.

### Cell Treatment

Gm31629-targeted small interfering RNAs (siRNAs) were purchased. The siRNA was transfected using lipofectamine RNAiMAX (Invitrogen) according to the instructions. Knockdown efficiency was detected by qPCR 2 days after transfection. Adenovirus control (Ad-Scramble) and adenovirus *Hnscr* (Ad-*Hnscr*) were generated from Obio Co (Shanghai, China). A 12-well plate was seeded with 3T3-L1 cells at 5 × 104 cells/well overnight (70-80% confluency), then transfected with siRNA or adenovirus at an multiplicity of infection (MOI) of 7 for 2 days before induction of adipocyte differentiation. Cells were treated with TF2A (80 μmol) for 72 hours before induction of adipocyte differentiation.

After 2 to 3 rounds of induction, the fluid was replaced with growth medium. Then cells were treated with H-89 (25 μM; Beyotime) for 2 hours. Cells were collected for further analysis.

### Rectal Temperature and Infrared Imaging

The mice were placed in a temperature-controlled chamber (MMM Friocell, Germany) at 4 °C for 4 hours. The rectal temperature was measured with a rectal probe at a specified time after cold exposure. Body temperatures were read before and 2 hours after cold exposure using infrared thermography (T1010, FLIR). Infrared images were analyzed with FLIR-Tools-Software (version 6.4).

### Western Blot Analysis

The following antibodies were purchased from Cell Signaling Technology: anti-ATGL (#2439, 1:1000, RRID:AB_2167953), anti-Phospho-HSL (#4137,1:1000, RRID:AB_2135498), anti-HSL (#4107,1:1000, RRID:AB_2296900), and anti-Phospho-PKA (#9621,1:1000, RRID:AB_330304). The following antibodies were purchased from Abcam: anti-cAMP (ab76238,1:1000, RRID:AB_1523259) and anti-UCP1 (ab234430,1:1000, RRID:AB_2905638).

### Quantitative Real-Time PCR

Real-time PCR analysis was conducted as previously described (28, 29). The SYBR Green PCR Master Mix was purchased from Accurate Biotechnology (China). The primer pairs used for qRT-PCR are listed (Table 1). The relative mRNA levels of target genes were given by the 2^−ΔΔCt^ method using β-actin as an internal control.

**Table 1.** Primer pairs

Gene	Forward primer	Reverse primer
*Hnscr*	CTAAGCGAACTCGGGAGC	CACAGCAGGATTGATGGATG
*SCD1*	GCGATACACTCTGGTGCTCA	CCCAGGGAAACCAGGATATT
*GPAT*	CACACGAGCAGGAAAGATGA	GGACTGCATAGATGCTGCAA
*PPARγ*	CTGGCCTCCCTGATGAATAA	CGCAGGTTTTTGAGGAACTC
*ChREBP*	CCTCACTTCACTGTGCCTCA	ACAGGGGTTGTTGTCTCTGG
*SREBP1c*	GGAGCCATGGATTGCACATT	GGAAGTCACTGTCTTGGTTGTTGA
*PPARα*	AGAGCCCCATCTGTCCTCTC	ACTGGTAGTCTGCAAAACCAAA
*CPT1a*	ATCGTGGTGGTGGGTGTGATAT	ACGCCACTCACGATGTTCTTC
*CD36*	TGGTCAAGCCAGCTAGAAA	CCCAGTCTCATTTAGCCAC
*FAS*	GGGTCTATGCCACGATTC	TGTCCCATGTTGGATTTG
*FABP4*	AAGGTGAAGAGCATCATAACCCT	TCACGCCTTTCATAACACATTCC
*ADIPOQ*	TGTTCCTCTTAATCCTGCCCA	CCAACCTGCACAAGTTCCCTT
*Lpl*	GGGAGTTTGGCTCCAGAGTTT	TGTGTCTTCAGGGGTCCTTAG
*Slc27a1*	CGCTTTCTGCGTATCGTCTG	GATGCACGGGATCGTGTCT
*UCP1*	CTGGAATAGCGGCGTGCTT	AATAACACTGGACGTCGGGC
*ATG1*	TGCTGTGGTGGAGGAGAG	TGTTGGAAAGGGTGGTCATC
*HSL*	CGAGACAGGCCTCAGTGTGA	GAATCGGCCACCGGTAAAG

### Histological Analysis of Tissues

Paraformaldehyde-fixed and paraffin-embedded iWAT and BAT sections were examined histologically with hematoxylin and eosin (H&E) staining. Adipocyte diameters were quantified using ImageJ V1.42q (National Institutes of Health, Bethesda, MD).

### Adipocytes Oil Red O Staining

Primary iWAT adipocytes or 3T3-L1 cells that induce differentiation maturation were collected, the medium was discarded, and cells were fixed with 4% paraformaldehyde for 20 minutes at room temperature. The 4% paraformaldehyde was discarded and cells were immersed in 60% isopropanol for 5 minutes. Then the 60% isopropanol was discarded and cells were stained with Oil Red O.

### Glycerin Measurement

Glycerin was measured with a glycerin kit according to the manufacturer’s instructions. The glycerin kit was purchased from Elabscience Company (Wuhan, China).

### Statistical Analysis

All data are expressed as means ± SEM. The statistical significance of the differences between various treatments or groups was measured by either Student's t-test or analysis of variance (ANOVA) followed by the Bonferroni post-test. Data analyses were performed using GraphPad Prism 7.0. *P* < .05 was considered statistically significant.

## Results

### Identification of Adipose lncRNA *Hnscr* as a Metabolic Signature

Our previous study found that lncRNA *Hnscr* could regulate systematic aging (25) and system insulin resistance (24). Therefore, we wondered whether lncRNA *Hnscr* played a vital role in lipid metabolism in adipose tissues.

**Figure 1.**
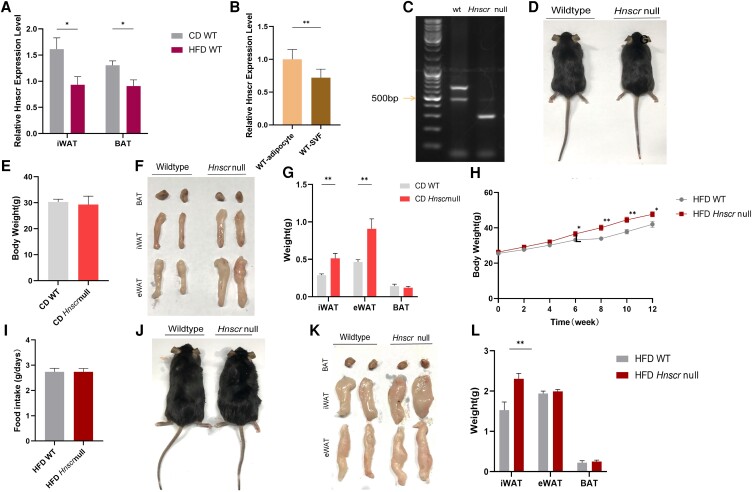
Identification of adipose lncRNA *Hnscr* as a metabolic signature. (A) RT-qPCR analysis of *Hnscr* mRNA in iWAT and BAT of 6-month-old mice fed NCD and HFD for 3 months (n = 6 mice). (B) RT-qPCR analysis of *Hnscr* mRNA in adipocytes and SVF of wildtype mice. (C) *Hnscr*-null mice and wildtype mice signature DNA band results. Appearance (D) and body weight (E) and of 6-month-old *Hnscr*-null and wildtype mice fed NCD (n = 6 mice). (F) Representative image of BAT, iWAT, and eWAT of 6-month-old *Hnscr*-null and wildtype mice fed with NCD (n = 6 mice). **(**G**)** Weight of iWAT, eWAT, and BAT of 6-month-old *Hnscr*-null and wildtype mice fed NCD (n = 6 mice). **(**H**)** Body weight of *Hnscr*-null and wildtype mice fed HFD during 3 months (n = 6 mice). **(**I**)** Food intake of 6-month-old *Hnscr*-null and wildtype mice fed HFD (n = 6 mice). **(**J**)** Appearance of 6-month-old *Hnscr*-null and wildtype mice fed HFD (n = 6 mice). **(**K**)** Representative image of BAT, iWAT, and eWAT of 6-month-old *Hnscr*-null and wildtype mice fed HFD. (L) Weight of iWAT, eWAT, and BAT of 6-month-old *Hnscr*-null and wildtype mice fed HFD (n = 6 mice). Statistical significance was calculated by 2-tailed Student's t test or 2-way ANOVA (*P < .05, **P < .01).

Firstly, we found that the expression level of *Hnscr* was decreased in iWAT and BAT in diet-induced obese mice (Fig. 1A). Furthermore, we detected the expression level of *Hnscr* in mature adipocytes and SVFs from iWAT of WT mice. The results showed that both mature adipocytes and SVFs expressed *Hnscr*, and mature adipocytes expressed higher levels of *Hnscr* than SVFs (Fig. 1B). To investigate the effect of *Hnscr* on lipolysis, we successfully constructed *Hnscr*-deficient (*Hnscr*-null) mice using TetraOne technology (Fig. 1C). Then the WT mice and *Hnscr*-null mice were fed NCD or HFD. For the NCD group, although the body weight displayed no significant difference between the WT mice and *Hnscr-*null mice (Fig. 1D and 1E), the weights of iWAT and epididymal white adipose tissue (eWAT) were increased in *Hnscr*-null mice (Fig. 1F and 1G).

In line with our expectation, the phenomena were more pronounced in the HFD groups. At 3 months of HFD feeding, *Hnscr*-null mice gained weight more rapidly than WT mice (Fig. 1H). We also examined the food intake of the 2 groups of mice and found that there was no significant difference (Fig. 1I). After 3 months of HFD intervention, *Hnscr*-deficient mice were fatter in appearance than WT mice (Fig. 1J). Although eWAT and BAT weights were not significantly different in *Hnscr*-null mice, iWAT weights were significantly increased (Fig. 1K and 1L). Together, the expression of *Hnscr* in adipose tissues decreased after HFD feeding, and *Hnscr* deletion correlated with body weight and fat weight, highlighting *Hnscr* as a metabolic feature of adipose tissue.

### *Hnscr* Knockout Promotes Lipogenesis and Decreases Lipolysis in iWAT

**Figure 2.**
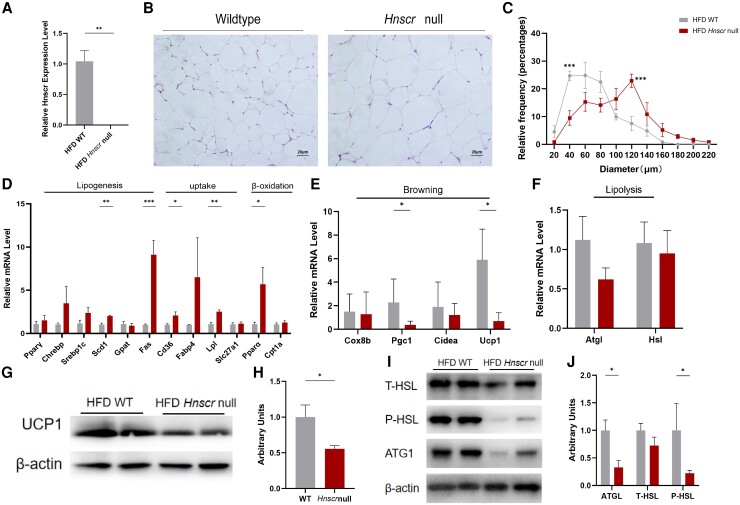
*Hnscr* knockout promotes lipogenesis and decreases lipolysis in iWAT. (A) mRNA levels of *Hnscr* in iWAT of 6-month-old *Hnscr*-null and wildtype mice fed HFD (n = 6 mice). (B) Representative H&E images of iWAT (n = 6 mice) of 6-month-old *Hnscr*-null and wildtype mice fed HFD. Scale bar: 20 μm. (C) Quantification of adipocyte diameter (n = 6 mice). (D) The mRNA levels of genes related to lipogenesis, fat uptake and β-oxidation in iWAT of 6-month-old *Hnscr*-null and wildtype mice fed with HFD (n = 6 mice). (E) The mRNA levels of genes related to browning in iWAT of 6-month-old *Hnscr*-null and wildtype mice fed HFD (n = 6 mice). (F) The mRNA levels of genes related to lipolysis in iWAT of 6-month-old *Hnscr*-null and wildtype mice fed HFD (n = 6 mice). (G, H) Protein level of UCP1 in iWAT of 6-month-old *Hnscr*-null and wildtype mice fed HFD (n = 6 mice). (I, J) Protein levels of ATGL and HSL phosphorylation in iWAT of 6-month-old *Hnscr*-null and wildtype mice fed HFD (n = 6 mice). Statistical significance was calculated by 2-tailed Student's t test or 2-way ANOVA (*P < .05, **P < .01, ***P < .001).

White adipose tissue is the largest triacylglycerol reservoir and free fatty acid supplier to other tissues (30). It is noteworthy that in obese people, subcutaneous lipolysis is reduced (31, 32). To verify the role of *Hnscr* in white adipose tissue, we extracted iWAT from *Hnscr*-null mice and WT mice fed with HFD. *Hnscr* expression was significantly lower in the iWAT of *Hnscr*-null mice (Fig. 2A). H&E staining demonstrated the iWAT of *Hnscr*-null mice contained a higher proportion of large adipocytes (Fig. 2B and 2C). RT-qPCR analysis revealed increased lipogenesis and fat uptake–related genes (Fig. 2D). Despite enrichment in some fat β-oxidation-related genes (Fig. 2D), the expression of browning and lipolysis-related genes decreased (Fig. 2E and 2F). Western blot (WB) analysis indicated that the expression of UCP1 decreased in iWAT of *Hnscr*-null mice (Fig. 2G and 2H). We thus asked if *Hnscr* knockout alters lipid metabolism in iWAT by evaluating the lipolysis enzyme ATGL and HSL phosphorylation that stimulate triglyceride hydrolysis. WB analysis showed that in the iWAT of *Hnscr*-null mice, the protein levels of ATGL and phosphorylation of HSL were reduced (Fig. 2I and 2J). The results show that the downregulation of *Hnscr* increases lipogenesis and decreases lipolysis in iWAT.

### *Hnscr* Knockout Decreases Lipolysis in BAT and Impairs Thermogenesis

**Figure 3.**
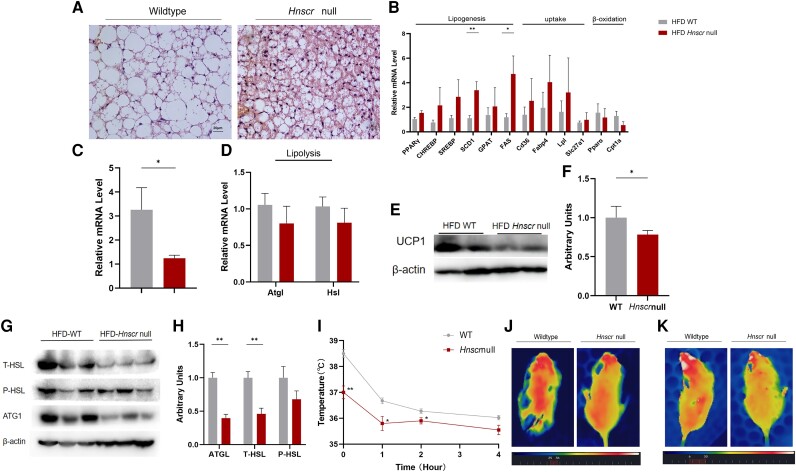
*Hnscr* knockout impairs energy expenditure and thermogenesis. (A) Representative H&E images of BAT (n = 6 mice) of 6-month-old *Hnscr*-null and wildtype mice fed HFD. Scale bar: 100 μm. **(**B**)** The mRNA levels of genes related to lipogenesis, fat uptake, and β-oxidation in BAT of 6-month-old *Hnscr*-null and wildtype mice fed HFD (n = 6 mice). (C) The mRNA levels of UCP1 in BAT of 6-month-old *Hnscr*-null and wildtype mice fed HFD (n = 6 mice). (D) The mRNA levels of genes related to lipolysis in BAT of 6-month-old *Hnscr*-null and wildtype mice fed HFD (n = 6 mice). (E, F) Protein level of UCP1 in BAT of 6-month-old *Hnscr*-null and wildtype mice fed HFD (n = 6 mice). (G, H) Protein levels of ATGL and HSL phosphorylation in BAT of 6-month-old *Hnscr*-null and wildtype mice fed HFD (n = 6 mice). (I) Rectal temperature of the mice (n = 4 mice). (J) Representative infrared thermal imaging of mice under room temperature (RT) (n = 4 mice). (K) Exposed to cold representative infrared thermal imaging of mice under cold condition (4 °C) for 2 hours (n = 4 mice). Statistical significance was calculated by 2-tailed Student's t test or 2-way ANOVA (*P < .05, **P < .01).

BAT is considered to be an important site of adaptive thermogenesis. The thermogenic activity of brown adipocytes can help resist obesity and metabolic diseases such as type 2 diabetes and dyslipidemia (33). Active BAT is present in adults, but its activity is impaired in obese patients (34). Therefore, we explored the role of *Hnscr* deletion in BAT. Compared with WT mice, H&E staining showed an increase in average adipocyte size due to a large amount of lipid accumulation in *Hnscr*-null mice (Fig. 3A). Similar to iWAT, there was a significant increase in genes associated with lipogenesis, accompanied by a trend toward increased fat uptake and decreased fat β-oxidation (Fig. 3B). UCP1 and lipolysis genes decreased in *Hnscr*-null mice in BAT (Fig. 3C and 3D). WB analysis revealed downregulation of UCP1 and lipolysis-related proteins in *Hnscr*-null mice (Fig. 3E-3H). Our results suggest that *Hnscr* knockout may promote fatty acid synthesis while reducing basal lipolysis in BAT during conditions of nutrient excess.

To determine the role of *Hnscr* in adaptive thermogenesis, mice were exposed to cold (4 °C). The rectal temperature of the mice exposed to cold (Fig. 3I) and infrared thermal imaging (under room temperature or 4 °C for 2 hours) (Fig. 3J and 3K) showed that *Hnscr*-null mice had a lower core body temperature than WT mice. These data demonstrate that *Hnscr* inhibition decreases lipolysis in BAT and impairs thermogenesis.

### *Hnscr* Knockdown in Adipocytes Decreases Lipolysis

**Figure 4.**
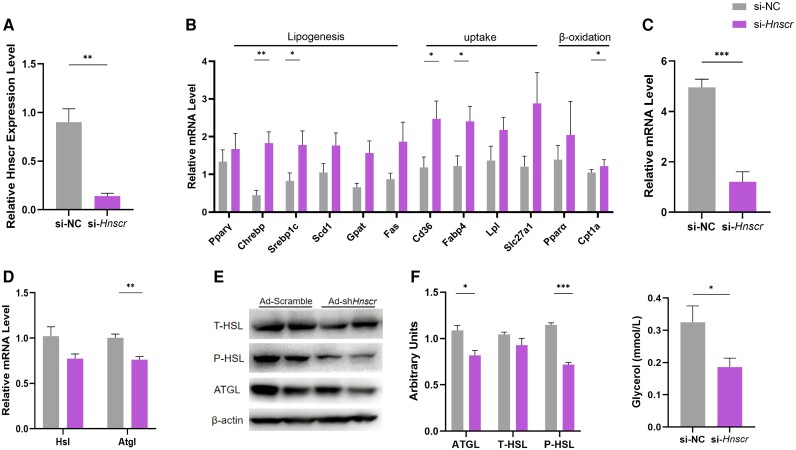
*Hnscr* knockdown in adipocytes decreases lipolysis. (A) The mRNA levels of *Hnscr* in primary iWAT adipocytes treated with si-*Hnscr* and si-NC (n = 6). (B) The mRNA levels of genes related to lipogenesis, fat uptake, and β-oxidation in primary iWAT adipocytes treated with si-*Hnscr* and si-NC (n = 6). (C) The mRNA levels of UCP1 in primary iWAT adipocytes treated with si-*Hnscr* and si-NC (n = 6). (D) The mRNA levels of genes related to lipolysis in primary iWAT adipocytes treated with si-*Hnscr* and si-NC (n = 6). (E, F) Protein levels of ATGL and HSL phosphorylation in primary iWAT adipocytes treated with Ad-*Hnscr* and Ad-scramble (n = 6). (G) Cell glycerol level. Statistical significance was calculated by 2-tailed Student's t test or 2-way ANOVA (*P < .05, **P < .01, ***P < .001).

To verify that differences in fat weight, cell size, and gene expression are a direct effect of *Hnscr* in adipose tissue, we transfected the *Hnscr* targeted siRNAs (si-*Hnscr*) and control siRNAs in primary adipocytes. The results show that si-*Hnscr* significantly inhibited the level of *Hnscr* in cells (Fig. 4A). Meanwhile, RT-qPCR analysis displayed that higher lipogenesis, fat uptake, and β-oxidation (Fig. 4B) coexisted with lower UCP1 and lipolysis (Fig. 4C and 4D) mRNA levels after *Hnscr* knockdown. Consistently, si-*Hnscr* treatment also decreased the expression of ATGL and p-HSL (Fig. 4E and 4F). Moreover, lipid measurement of cells also revealed that knockdown of *Hnscr* in adipocytes decreased glycerol level (Fig. 4G). Taken together, we concluded that *Hnscr* deficiency inhibits adipocytes lipolysis.

### Overexpression of *Hnscr* Facilitates Lipolysis of Adipocytes

**Figure 5.**
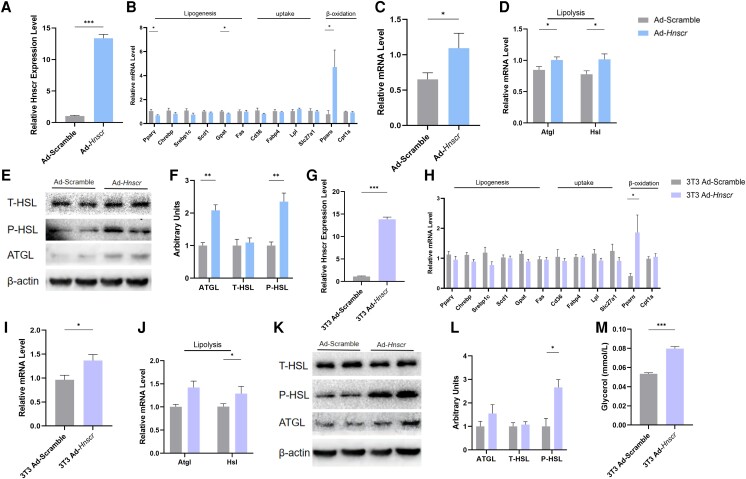
*Hnscr* promotes adipocyte lipolysis expression. (A) mRNA levels of *Hnscr* in primary iWAT adipocytes treated with Ad-*Hnscr* and Ad-scramble (n = 6). (B) mRNA levels of genes related to lipogenesis, fat uptake, and β-oxidation in primary iWAT adipocytes treated with Ad-*Hnscr* and Ad-scramble (n = 6). (C) mRNA levels of UCP1 in primary iWAT adipocytes treated with Ad-*Hnscr* and Ad-scramble (n = 6). (D) The mRNA levels of genes related to lipolysis in primary iWAT adipocytes treated with Ad-*Hnscr* and Ad-scramble (n = 6). (E, F) Protein levels of ATGL and HSL phosphorylation in primary iWAT adipocytes treated with Ad-*Hnscr* and Ad-scramble (n = 6). (G) The mRNA levels of *Hnscr* in 3T3-L1 cells treated with Ad-*Hnscr* and Ad-scramble (n = 6). (H) mRNA levels of genes related to lipogenesis, fat uptake, and β-oxidation in 3T3-L1 cells treated with Ad-*Hnscr* and Ad-scramble (n = 6). (I) mRNA levels of UCP1 in 3T3-L1 cells treated with Ad-*Hnscr* and Ad-scramble (n = 6). (J) mRNA levels of genes related to lipolysis in 3T3-L1 cells treated with Ad-*Hnscr* and Ad-scramble (n = 6). (K, L) Protein levels of ATGL and HSL phosphorylation in 3T3-L1 cells treated with Ad-*Hnscr* and Ad-scramble (n = 6). (M) Cell glycerol level in 3T3-L1 cells. Statistical significance was calculated by 2-tailed Student's t test or 2-way ANOVA (*P < .05, **P < .01, ***P < .001).

To explore the effect of *Hnscr* in adipocytes, primary adipocytes were isolated and transfected with an *Hnscr*-expressing adenovirus (Ad-*Hnscr*). As expected, the expression of *Hnscr* was dramatically increased compared with the control group (Fig. 5A). Overexpression of *Hnscr* downregulated mRNA levels of genes involved in lipogenesis in differentiated primary adipocytes (Fig. 5B). Moreover, the levels of β-oxidation, and thermogenic and lipolysis-related genes were elevated in the *Hnscr* overexpression group (Fig. 5B-5D). WB analysis confirmed the protein level that *Hnscr* could promote lipolysis (Fig. 5E and 5F). These data indicate that overexpression of *Hnscr* facilitates lipolysis of primary adipocytes.

3T3-L1 preadipocytes are clonally isolated sublineages from 3T3 cells (Swiss albino) (35, 36). The 3T3-L1 cell line can be stable for passage and has reasonable specificity for differentiation to adipocytes, so it has become an internationally recognized cell model for studying adipose metabolism (37, 38). Therefore, we transfected it with a *Hnscr*-expressing adenovirus in 3T3-L1 cells (Fig. 5G). Consistent with the situation in primary adipocytes, overexpression of *Hnscr* in differentiated 3T3-L1 cells produced a trend toward downregulation of mRNA levels of genes involved in lipogenesis and fat uptake.(Fig. 5H), accompanied by upregulating β-oxidation, thermogenic, and lipolysis genes (Fig. 5H and 5J). WB analysis also indicated that the levels of lipolysis-related proteins increased (Fig. 5K and 5L). Cell lipid measurement revealed that overexpression of *Hnscr* in adipocytes increased the glycerol level (Fig. 5M). In conclusion, these combined results show that *Hnscr* promotes lipolysis in adipocytes.

### TF2A Treatment In Vitro Promotes Lipolysis of Adipocytes

**Figure 6.**
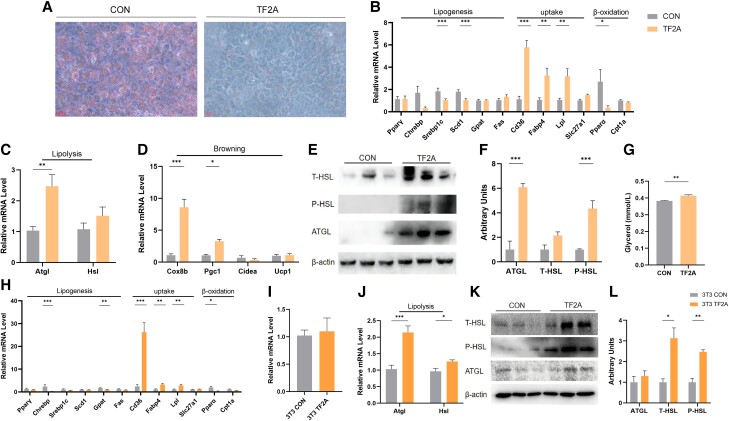
Administration of TF2A promoted lipolysis of adipocytes. (A) Oil red staining of representative differentiated primary iWAT adipocytes administrated TF2A or vehicle (n = 6) at day 7. Scale bar: 100 μm. (B) mRNA levels of genes related to lipogenesis, fat uptake, and β-oxidation in primary iWAT adipocytes administrated TF2A or vehicle (n = 6). (C) mRNA levels of genes related to lipolysis in primary iWAT adipocytes administrated TF2A or vehicle (n = 6). (D) mRNA levels of genes related to browning in primary iWAT adipocytes administrated TF2A or vehicle (n = 6). (E, F) Protein levels of ATGL and HSL phosphorylation in primary iWAT adipocytes administrated TF2A or vehicle (n = 6). (G) Cell glycerol level in primary iWAT adipocytes administrated TF2A or vehicle (n = 6). (H) mRNA levels of genes related to lipogenesis, fat uptake, and β-oxidation in 3T3-L1 cells administrated TF2A or vehicle (n = 6). (I) mRNA level of UCP1 in 3T3-L1 cells administrated TF2A or vehicle (n = 6). (J) mRNA levels of genes related to lipolysis in 3T3-L1 cells administrated TF2A or vehicle (n = 6). (K, L) Protein levels of ATGL and HSL phosphorylation in 3T3-L1 cells administrated TF2A or vehicle (n = 6). Statistical significance was calculated by 2-tailed Student's t test or 2-way ANOVA (*P < .05, **P < .01, ***P < .001).

Previously, we found that TF2A, a small, naturally occurring compound, could mimic the activity of *Hnscr* (25). To confirm the role of *Hnscr* in regulating lipid metabolism, mice primary adipocytes and 3T3-L1 cells were incubated with TF2A. Oil Red O staining results indicated that administration of TF2A inhibited lipid accumulation in adipocytes (Fig. 6A). The levels of lipogenesis genes in the TF2A groups were also decreased (Fig. 6B and Fig. 6H). Although increased expression of uptake genes and reduced expression of β-oxidation genes (Fig. 6B and Fig. 6H) were found in the TF2A groups, lipolysis and browning-related genes were significantly upregulated (Fig. 6C and 6D and Fig. 6I and 6J).

Further verification by WB experiments showed that the lipolytic capacity of primary adipocytes and 3T3-L1 cells was augmented in the TF2A groups (Fig. 6E and F and Fig. 6K and 6L). Cells lipid measurement revealed that treatment with TF2F in adipocytes increased the glycerol level (Fig. 6G). In summary, the *Hnscr* mimic, TF2A, exerts a beneficial effect on lipid metabolism of adipose tissue by promoting lipolysis, indicating its therapeutic potential.

### *Hnscr* Activates the cAMP/PKA Signaling Pathway to Promote Lipolysis in Adipocytes

**Figure 7.**
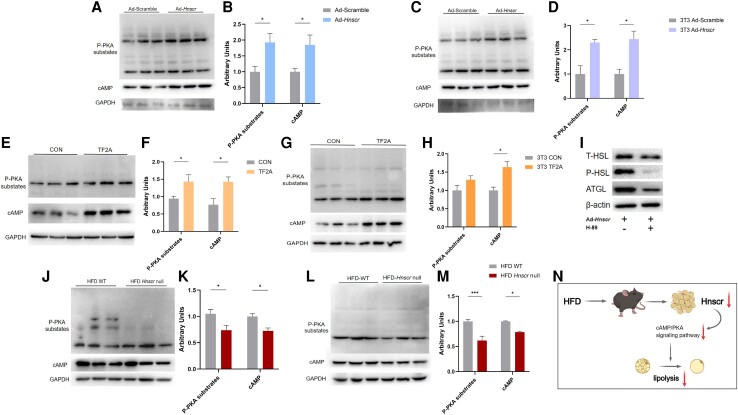
*Hnscr* promotes adipose lipolysis expression by mediating the cAMP/PKA signaling pathway. (A, B) Protein levels of cAMP and PKA phosphorylation in primary iWAT adipocytes treated with Ad-*Hnscr* and Ad-scramble (n = 6). (C, D) Protein levels of cAMP and PKA phosphorylation in 3T3-L1 cells treated with Ad-*Hnscr* and Ad-scramble (n = 6). (E, F) Protein levels of cAMP and PKA phosphorylation in primary iWAT adipocytes administrated TF2A or vehicle (n = 6). (G, H) Protein levels of cAMP and PKA phosphorylation in 3T3-L1 cells administrated TF2A or vehicle (n = 6). (I) Protein levels of ATGL and HSL phosphorylation in overexpress *Hnscr* 3T3-L1 cells treated with or without H-89. (J, K) Protein levels of cAMP and PKA phosphorylation in iWAT of *Hnscr*-null mice treated with HFD (n = 6 mice). (L, M) Protein levels of cAMP and PKA phosphorylation in BAT of *Hnscr*-null mice treated with HFD (n = 6 mice). (N) Schematic diagram of *Hnscr*-mediated regulation of lipolysis. Statistical significance was calculated by 2-tailed Student's t test or 2-way ANOVA (*P < .05, ***P < .001).

The cAMP/PKA signaling pathway is a well-established mechanism for activating lipolysis in adipose tissue (21). In our previous study, the cAMP/PKA signaling generally activated lipolysis by stimulating HSL phosphorylation in peripheral white adipose tissues (23). Therefore, we investigated whether *Hnscr* promotes adipocyte lipolysis by activating the cAMP/PKA signaling pathway. WB analysis indicated *Hnscr* overexpression promoted protein levels of cAMP and phosphorylation of PKA in both primary adipocytes and 3T3-L1 cells (Fig. 7A-7D). Furthermore, the expression levels of cAMP and phosphorylated forms of PKA were also significantly increased in the TF2A treatment groups (Fig. 7E-7H). We pretreated adipocytes overexpressing *Hnscr* with H-89, a pharmacological PKA inhibitor. The results showed significantly decreased lipolysis-related proteins (Fig. 7I).

To confirm our results, we detected the level of cAMP/PKA signaling in vivo. Compared with WT mice, the protein levels of cAMP and phosphorylation PKA were significantly decreased in iWAT and BAT of *Hnscr*-null mice (Fig. 7J-7M). Therefore, these findings clarify that *Hnscr* regulates adipose tissue lipolysis by activating the cAMP/PKA signaling pathway (Fig. 7N).

## Discussion

It has been shown that HFD-induced obesity significantly decreases the proportion of mature adipocytes, and substantially increases the size of mature fat cells (adipocyte hypertrophy) and adipose tissue weight (39). One of the most important observations in this study was the finding of decreased levels of lipolysis in adipose tissues of *Hnscr*-null mice, which was particularly evident after excess nutrition. We first found downregulation of lncRNA *Hnscr* expression in iWAT and BAT of the HFD-induced obese mice. Since *Hnscr* has been shown to mediate systematic glucose metabolism in previous studies (24), we constructed *Hnscr*-null mice to investigate its role in lipid metabolism. Past studies have shown that a critical feature of obesity is excess storage of triglycerides in adipose tissue through adipocyte hyperplasia (increased number) or hypertrophy (increased volume), or even both. It is generally believed that adipocyte hypertrophy occurs before adipocyte hyperplasia and is the primary mechanism of fat mass expansion (40). In this study, H&E staining showed that loss of *Hnscr* resulted in mice adipocyte hypertrophy. In iWAT and BAT, *Hnscr* deletion led to increased lipogenesis and fat uptake in adipose tissues, but the expression of thermogenic genes such as UCP1 and lipolytic genes was downregulated.

Lipolysis is described as the metabolic process of catabolism of triacylglycerols stored in cellular lipid droplets (41). Abnormal lipolysis may be associated with lipodystrophy, hyperlipidemia, or obesity (42, 43). ATGL and HSL are responsible for more than 90% of triglyceride hydrolysis in adipose tissue. It is found that ATGL is the essential lipase for the hydrolysis of triglycerides to diglycerides, which HSL catalyzes into monoglycerides and glycerol (6, 7). We explored the role of *Hnscr* in in vitro experiments and found that overexpression of *Hnscr* in adipocytes inhibited lipogenesis and promoted lipolysis. Furthermore, our results also showed that although the intervention of adipocytes with TF2A resulted in increased expression of genes related to lipogenesis and uptake, there were significantly increased levels of browning and lipolysis.

The cAMP/PKA signaling pathway is a recognized mechanism for activating lipolysis in adipose tissue (23). The results confirmed that *Hnscr* regulated adipose lipid metabolism by mediating the cAMP/PKA signaling pathway. Overexpression of *Hnscr* in adipocytes increased the protein levels of cAMP/PKA signaling. Moreover, TF2A can mimic the action of *Hnscr* and promote cAMP/PKA activation. We also verified in *Hnscr*-null mice that *Hnscr* knockout downregulated the cAMP/PKA signaling activity in adipose tissues.

Starting from the discovery of decreased *Hnscr* expression in iWAT and BAT of obese mice, we constructed *Hnscr*-null mice to explore the role of *Hnscr* in adipose tissue. Meanwhile, we also found that overexpression of *Hnscr* promoted lipolysis in adipocytes. Mechanistically, *Hnscr* regulated lipid metabolism by activating the cAMP/PKA signaling pathway. We also intervened in adipocytes with TF2A, a compound that could mimic the activity of *Hnscr* to promote lipolysis (25). Future works should identify the potential PKA phosphorylation sites on *Hnscr* and their impact on its function. The reasons for the contradictory relationship between TF2A in fat uptake and lipolysis were also studied. This study revealed that *Hnscr* could regulate lipid metabolism in obese mice by mediating adipocyte lipolysis, which could become a new therapeutic target for obesity.

## Financial Support

This work was supported by grants from the National Natural Science Foundation of China (no. 82000811).

## Data Availability

Original data generated and analyzed during this study are included in this published article or in the data repositories listed in References.

## Abbreviations

ANOVA: analysis of variance
ATGL: adipose triglyceride lipase
BAT: brown adipose tissue
cAMP: cyclic adenosine monophosphate
DMEM: Dulbecco’s modified Eagle’s medium
eWAT: epididymal white adipose tissue
H&E: hematoxylin and eosin
HFD: high-fat diet
HSL: hormone-sensitive lipase
iWAT: inguinal white adipose tissue
lncRNA: long noncoding RNA
NCD: normal chow diet
PCR: polymerase chain reaction
PKA: protein kinase A
siRNA: small interfering RNA
SVF: stromal vascular fraction
TF2A: theaflavin 3-gallate
WB: Western blot
WT: wildtype
